# The effect of DNA methylation on bumblebee colony development

**DOI:** 10.1186/s12864-021-07371-1

**Published:** 2021-01-22

**Authors:** María I. Pozo, Benjamin J. Hunt, Gaby Van Kemenade, Jose M. Guerra-Sanz, Felix Wäckers, Eamonn B. Mallon, Hans Jacquemyn

**Affiliations:** 1 KU Leuven, Biology Department, Plant Population and Conservation Biology, B-3001 Heverlee, Belgium; 2 Department of Genetics and Genome Biology, University of Leicester, Leicester, LE1 7RH United Kingdom; 3 Biobest Group, Research and Development, B-2260 Westerlo, Belgium; 4 Independent Researcher, La Mojonera, 04745 Almeria, Spain

**Keywords:** DNA methylation, Social insects, Epigenetics, Colony development

## Abstract

**Background:**

Although around 1% of cytosines in bees’ genomes are known to be methylated, less is known about methylation’s effect on bee behavior and fitness. Chemically altered DNA methylation levels have shown clear changes in the dominance and reproductive behavior of workers in queen-less colonies, but the global effect of DNA methylation on caste determination and colony development remains unclear, mainly because of difficulties in controlling for genetic differences among experimental subjects in the parental line. Here, we investigated the effect of the methylation altering agent decitabine on the developmental rate of full bumblebee colonies. Whole genome bisulfite sequencing was used to assess differences in methylation status.

**Results:**

Our results showed fewer methylated loci in the control group. A total of 22 CpG loci were identified as significantly differentially methylated between treated and control workers with a change in methylation levels of 10% or more. Loci that were methylated differentially between groups participated in pathways including neuron function, oocyte regulation and metabolic processes. Treated colonies tended to develop faster, and therefore more workers were found at a given developmental stage. However, male production followed the opposite trend and it tended to be higher in control colonies.

**Conclusion:**

Overall, our results indicate that altered methylation patterns resulted in an improved cooperation between workers, while there were no signs of abnormal worker dominance or caste determination.

**Supplementary Information:**

The online version contains supplementary material available at 10.1186/s12864-021-07371-1.

## Background

The phenotype of an individual is determined, ultimately, by the context-driven interpretation of a given DNA sequence [1]. DNA methylation, of which the most common example is the addition of a methyl group to a cytosine, is a reversible biological process that can change the activity of a DNA segment {Citation} [2] and lead to phenotypic plasticity in modular organisms such as plants [3], or induce reversible and quick adaptation to stress conditions in unicellular fungi [4]. In social insects, where genetic relatedness affects cooperation and reproductive behavior within colonies [5], DNA methylation has been shown to affect important phenotypic features such as caste differentiation and worker reproductive behavior [6, 7].

Genome-wide methylation levels are highly variable and differ substantially between major taxonomical groups. In mammals for example, about 70% of CpG dinucleotides are methylated in somatic cells [8], while the genome of most plants, invertebrates, fungi, or protists shows “mosaic” methylation patterns, where only specific genomic elements are targeted and distinct patterns of methylated and unmethylated domains can be discerned [9, 10]. In insects, DNA methylation levels are often low (less than 1%), but they generally concentrate in gene-coding regions [11]. While DNA methylation at gene promoter regions suggests gene silencing as the main function, as proposed for mammals [12], methylation within gene-coding regions suggests a role in alternative splicing [13]. However, in the particular case of insects such as bumblebees, differentially expressed genes contain lower levels of methylation compared to non-differentially expressed genes, which further indicates that DNA methylation in bumblebees is more related to gene expression than to an alteration of gene function [14, 15].

One of the most defining characteristic of insect societies is the reproductive division of labor, where workers usually do not produce offspring in the presence of a queen [16]. The prevailing theory that explains such ‘altruistic’ behavior is inclusive fitness [17, 18], which states that workers will be selected to nurse their mother’s offspring rather than investing time and energy in their own progeny. In bumblebees, altruistic worker behavior can be explained by the higher relatedness of the female sisters, who would share ¾ of their genome, compared to the relatedness of the workers to their own male progeny. The annual colony life cycle of a bumblebee is therefore divided into a cooperative phase, when female workers are produced while the queen has absolute reproductive dominance, and a highly aggressive competition phase later in the season when the workers and queen compete over male production [19]. Bumblebee queens mate only once, implying that all workers are full sisters and that their genomes therefore have all the necessary information to become “dominant” and show reproductive behavior. However, if the queen dies or is removed, unmated workers can differentiate into reproductive and non-reproductive sub-castes by both their ovary development and aggressive behavior [20].

The genomes of queen-less reproductive workers and queen-less non-reproductive workers have been shown to differ in methylation levels [7], suggesting that worker reproductive behavior may be determined, and inherited, by epigenetic factors. Moreover, queen-less workers whose genomes had been experimentally altered by using an inhibitor of DNA methyl-transferase were more aggressive and more likely to develop ovaries compared with control queen-less workers [7], indicating that DNA methylation is important in this highly plastic reproductive division of labor. However, these results also indicate that variation in DNA methylation levels could affect overall colony development: if workers with low DNA methylation levels gain dominance and are more keen to reproduce, the resulting colonies would show an earlier disruption of the cooperation phase, which in turn would lead to a higher production of males and a decreased production of queens [19], but see [21]. However, the reproductive behavior of bumblebee workers, when separated from the queen under laboratory conditions, can be expected to differ from those in colonies where the founder queen remains present. Most experiments studying epigenetic effects on worker behavior used micro-colonies of full sisters that were obtained and kept separately from single mated *Bombus* queens [22]. While this ensures that all individuals have the same genotype, the absence of the queen is a rather unnatural situation that may have a profound impact on the results.

Caste determination is another feature of social insects that can be affected by epigenetic factors. Previous work on honeybees has shown that changes in methylation levels are involved in the switch between workers and queens [23]. The comparison of larval heads between queens and workers of honeybees show a total of 2399 genes with significant differences of methylation [24]. In addition, substantial differentially methylated genes were found among different castes in the termite *Zootermopsis nevadensis* [25] and the ant *Camponotus floridanus* [26]. However, no association between caste and methylation has been found in some primitive wasps, such as *Polistes* spp. ([27]). Recently, [15] found differences in methylation levels between reproductive castes of bumblebee workers, with some differentially methylated genes involved in behavior and reproductive processes. Their results also showed high inter-colony variance in methylation levels, suggesting that different couples of queens and males transmit different methylomes to their progeny [28–30], which in turn will lead to developmental differences at the colony level [31]. DNA methylation could also be involved in worker vs. (daughter) queen development by fertilized eggs in bumblebees.

In this study, we tested the hypothesis that DNA methylation had a significant effect on colony development of the bumblebee *Bombus terrestris*. By experimentally exposing a pure genetic line of *B. terrestris* founders to the methylation disruptor decitabine through sugar water provisions, we first investigated the effect of DNA methylation on the developmental fate of larvae, and how this affected colony development. Second, to identify the specific loci that were affected by the addition of decitabine and the biological pathways these genes were involved in, brain tissue samples were collected from adult workers to be examined for DNA methylation at single base resolution using whole genome bisulfite sequencing (WGBS).

## Results

### Temporal succession of main colony events

We obtained 6/6 and 5/6 developed colonies in control and treated queens, respectively. The time needed to lay the first eggs or to produce the first pupae did not differ significantly between founder queens that were assigned to the different treatment levels (W = 15.0, *P* = 1; W = 12.5, *P* = 0.7112 for eggs and pupae, respectively). Correspondingly, the number of days needed to produce the first workers in a colony also did not differ significantly between treatments (W = 12.5, *P* = 0.7138), nor did date of first male appearance (W = 14, *P* = 0.926).

### Colony size and production of males

**Fig. 1:**
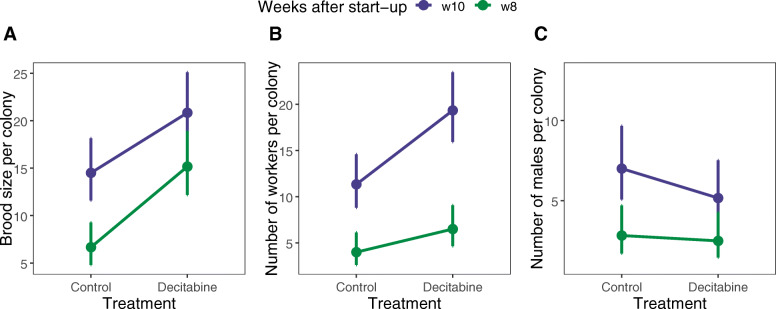
(**a**) Average brood size (sum of egg cups and larvae), (**b**) average number of workers and (**c**) average number of males per colony for control and decitabine-treated colonies, counted 8 and 10 weeks after start-up. Symbol depict ls-means, plotted at the original scale, and vertical lines show 95% confidence intervals

Overall, colonies supplemented with decitabine had a significantly higher brood size than control colonies (χ^2^ = 23.36, *P* < 0.001, Fig. 1a). In agreement with the end of the cooperative phase of the colony, differences were more pronounced when colonies were counted 8 weeks after colony start-up (Z = − 4.33, *P* < 0.0001), although they remained significant when colonies were examined two weeks later (Z = − 2.60, *P* = 0.046, Fig. 1a). Larval mortality did not differ among control and treated colonies (χ^2^ = 0.14, *P* = 0.705). However, queens treated with decitabine were more active at laying eggs before the competition point, as indicated by the significantly higher number of egg cups in treated colonies at week 8 (χ^2^ = 24.12, *P* < 0.001). Correspondingly, treatment also positively affected worker production (χ^2^ = 16.25, *P* < 0.001, Fig. 1b). This effect was consistent at both assessment weeks, although for this parameter developmental differences accumulated over time, yielding significant differences for worker production for the last counting week only (Fig. 1b, z = − 1.871, *P* = 0.240, and Z = − 3.497, *P* = 0.003 for weeks 8 and 10 after colony start-up, respectively).

The administration of decitabine tended to decrease the number of males produced after the competition point (week 10, Fig. 1c). However, the overall effect of the treatment on male production did not reach statistical significance (χ^2^ = 1.614, *P* = 0.204).

### Worker reproductive behaviour

Random dissections at week 8 showed developed ovaries as the most observed stage of worker reproductive status at that point of colony development, and therefore it was observed in 7/10 (control) and 8/10 of the dissected workers (treated colonies). Remaining specimens showed either incipient (1/10 of treated colonies, 0/10 in control) or no ovary development (3/10 and 2/10 for control and treated workers, respectively). The frequency of occurrence of each ovary status category did not differ among treatments (odds ratio = 0.599, *P* = 1; odds ratio = 0.000, P = 1; and odds ratio = 3.611, *P* = 0.582 for developed, incipient and no developed ovaries, respectively).

Egg dumping behaviour was observed in 2/6 (control) and 1/5 (treated) of the colonies, which led to similar occurrence of this behaviour among experimental groups (odds ratio = 1.879, *P* = 1).

### Methylation differences

Overall, the mean mapping efficiency was (mean ± SD) 39.5 ± 2.9%. The percentage of methylated CpG’s in control conditions was a mean of 0.5 ± 0.2% (mean ± SD). Similar levels were also found in non-CpG methylation contexts, a mean of 0.4 ± 0.1% of CHG’s were methylated and 0.4 ± 0.1% CHH’s (mean ± SD, referring ‘H’ to any base other than guanine). For the set of workers treated with decitabine, methylation rates were almost two-fold, with an average of 0.9 ± 0.1, 0.7 ± 0.1, and 0.8 ± 0.1 (mean ± SD), for CpG, CHG and CHH methylation types, respectively.

The current *B. terrestris* annotation file (Refseq accession no. GCF_000214255.1) was used as a reference to calculate the proportion of methylated reads for the C-context that were located in annotated loci. Variation in CpG methylation between treatment levels was much higher than variation caused by colonies (Fig. S1 A,B). Workers from treated colonies showed higher percentages of loci with significant levels of methylation (0.08% vs 0.04%, as group average, Table 1). Of the significantly methylated loci, methylation fraction (number of C reads / total number of reads) appears to be similar between treated and control groups. (Table 1).

**Table 1:** Overview of mapping efficiency, methylation rates in CpG, CHG and CHH contexts, number of significantly methylated loci and methylation fraction per loci for the 14 workers subjected to WGBS

Treatment	Colony	Worker id	Mapping efficiency	Methylation rate (CpG)	Methylation rate (CHG)	Methylation rate (CHH)	N of significantly methylated loci	Total Loci	%	Methylation fraction (numCs/CT)
D	5	w1	44.4	1.00	0.80	0.90	3044	3,534,447	0.09%	0.44
D	1	w3	43	0.90	0.71	0.80	2835	3,515,033	0.08%	0.45
D	1	w4	41.7	0.79	0.62	0.70	1969	2,720,084	0.07%	0.47
D	1	w5	38.5	0.88	0.71	0.80	1478	2,658,036	0.06%	0.41
D	5	w2	43.1	0.86	0.69	0.77	2664	2,889,178	0.09%	0.49
D	5	w4	38.4	0.88	0.71	0.80	1706	2,723,798	0.06%	0.44
D	5	w5	35.3	0.86	0.69	0.78	1921	2,321,704	0.08%	0.46
C	5	w5	39.4	0.46	0.35	0.40	1073	2,248,205	0.05%	0.44
C	2	w2	36.2	0.46	0.36	0.42	1270	2,741,953	0.05%	0.43
C	2	w1	38.9	0.41	0.32	0.37	1145	3,463,111	0.03%	0.42
C	2	w4	35.3	0.42	0.32	0.38	1006	2,592,099	0.04%	0.43
C	5	w2	36.8	0.42	0.32	0.38	855	2,849,397	0.03%	0.44
C	5	w3	39	0.43	0.33	0.38	935	2,063,422	0.05%	0.41
C	5	w4	42.6	0.83	0.66	0.74	1918	2,864,878	0.07%	0.45

A total of 22 loci (methylation differences > 10%, *q* = 0.05) showed significant methylation differences in the CpG context between treated and control groups. Of these, 20 differentially methylated loci between treated and control bees that could be successfully annotated. These loci were associated with GO enrichment for terms including regulation of oogenesis, oocyte mRNA localisation, neuronal function and various metabolic processes (Table 2). No differentially methylated loci were identified in a non-CpG context.

**Table 2:** GO enrichment analysis- biological processes (BP) terms of the set of differentially methylated loci for treated and untreated workers at 10% methylation difference threshold

GOBPID	*P*value	Odds Ratio	Count	Size	Term
GO:0030423	0.002	Inf	1	1	targeting of mRNA for destruction involved in RNA interference
GO:0007274	0.002	31.976	2	35	neuromuscular synaptic transmission
GO:0042062	0.004	496.625	1	2	long-term strengthening of neuromuscular junction
GO:0055083	0.006	248.281	1	3	monovalent inorganic anion homeostasis
GO:0072505	0.006	248.281	1	3	divalent inorganic anion homeostasis
GO:0072506	0.006	248.281	1	3	trivalent inorganic anion homeostasis
GO:0007415	0.006	248.281	1	3	defasciculation of motor neuron axon
GO:0030643	0.006	248.281	1	3	cellular phosphate ion homeostasis
GO:0030002	0.009	165.500	1	4	cellular anion homeostasis
GO:1990034	0.009	165.500	1	4	calcium ion export across plasma membrane
GO:0008038	0.010	14.791	2	73	neuron recognition
GO:0044205	0.011	124.109	1	5	‘de novo’ UMP biosynthetic process
GO:0043102	0.011	124.109	1	5	amino acid salvage
GO:0032728	0.011	124.109	1	5	positive regulation of interferon-beta production
GO:0019509	0.011	124.109	1	5	L-methionine salvage from methylthioadenosine
GO:0006796	0.011	3.750	7	1257	phosphate-containing compound metabolic process
GO:0071265	0.013	99.275	1	6	L-methionine biosynthetic process
GO:0048790	0.013	99.275	1	6	maintenance of presynaptic active zone structure
GO:0006309	0.015	82.719	1	7	apoptotic DNA fragmentation
GO:0031054	0.015	82.719	1	7	pre-miRNA processing
GO:0006207	0.017	70.893	1	8	‘de novo’ pyrimidine nucleobase biosynthetic process
GO:0046049	0.017	70.893	1	8	UMP metabolic process
GO:0007026	0.017	70.893	1	8	negative regulation of microtubule depolymerization
GO:0007482	0.017	70.893	1	8	haltere development
GO:0031629	0.017	70.893	1	8	synaptic vesicle fusion to presynaptic active zone membrane
GO:0046475	0.019	62.023	1	9	glycerophospholipid catabolic process
GO:0009174	0.019	62.023	1	9	pyrimidine ribonucleoside monophosphate biosynthetic process
GO:0006921	0.021	55.125	1	10	cellular component disassembly involved in execution phase of apoptosis
GO:0030422	0.021	55.125	1	10	production of siRNA involved in RNA interference
GO:0051693	0.021	55.125	1	10	actin filament capping
GO:0009129	0.023	49.606	1	11	pyrimidine nucleoside monophosphate metabolic process
GO:0007317	0.025	45.091	1	12	regulation of pole plasm oskar mRNA localization
GO:0000097	0.025	45.091	1	12	sulfur amino acid biosynthetic process
GO:0072499	0.027	41.328	1	13	photoreceptor cell axon guidance
GO:0016081	0.027	41.328	1	13	synaptic vesicle docking
GO:0030834	0.027	41.328	1	13	regulation of actin filament depolymerization
GO:0006555	0.029	38.144	1	14	methionine metabolic process
GO:0046112	0.029	38.144	1	14	nucleobase biosynthetic process
GO:0046132	0.029	38.144	1	14	pyrimidine ribonucleoside biosynthetic process
GO:1903779	0.029	38.144	1	14	regulation of cardiac conduction
GO:0006206	0.034	33.050	1	16	pyrimidine nucleobase metabolic process
GO:0009067	0.034	33.050	1	16	aspartate family amino acid biosynthetic process
GO:0009220	0.034	33.050	1	16	pyrimidine ribonucleotide biosynthetic process
GO:0002028	0.036	30.980	1	17	regulation of sodium ion transport
GO:0051290	0.036	30.980	1	17	protein heterotetramerization
GO:0043242	0.038	29.154	1	18	negative regulation of protein complex disassembly
GO:1905879	0.040	27.531	1	19	regulation of oogenesis
GO:0006213	0.040	27.531	1	19	pyrimidine nucleoside metabolic process
GO:0031050	0.040	27.531	1	19	dsRNA processing
GO:0032272	0.044	24.772	1	21	negative regulation of protein polymerization
GO:0008333	0.048	22.514	1	23	endosome to lysosome transport

## Discussion

Social insects are among the most successful on Earth, due to their ability to divide tasks among castes and to cooperate (see [32] and references therein). In many cases, individuals with different functions within the colony are genetically identical, which immediately raises the question whether and how epigenetic regulation contributes to this phenotypic differentiation. Previous studies on honeybees have shown that DNA methylation affects larval differentiation into worker-queen castes [23, 24]. In this study, we have experimentally manipulated the methylomes of founder queens of *B. terrestris* belonging to the same single parental line by using decitabine in order to infer the role of DNA methylation on phenotypic features such as caste determination and reproductive behaviour. The resulting colonies were monitored during ten weeks and their offspring was compared with colonies receiving a control treatment. Furthermore, the efficacy of the treatment was tested for a random subset of workers by analysing their methylomes using WGBS (see graphical summary in Supplementary Material).

### Effects of decitabine on colony development

Chemically-induced DNA demethylation has been already proven to contribute to phenotypic variation in insects [33, 34]. In this study, we used full colonies (queen-right) instead of queen-less micro-colonies of bumblebee workers to assess the effect of altered methylation on colony development and caste determination, which is impossible to assess when using unfertilized workers. Our results showed that colonies that received a continuous supply of decitabine grew better than control colonies receiving no decitabine. In absence of genetic differences among queen founders, and considering that there were no temporal differences for the succession of main colony events, such an effect was mainly due to a higher egg-laying activity by the queen itself, combined with the higher maturation success of the queen brood. Gene ontology for the set of differentially methylated genes in the queen female offspring included oogenesis regulation and oocyte mRNA localization, consistent with the suggestion that founder queens got an enlarged progeny from their set of eggs. Moreover, decitabine is supposed to have no effect on post-mitotic adult specimens, such as the founder queen in this particular setup (7).

In our experiment, we did not analyse direct effects of decitabine on the queen methylomes, as this would have precluded the completion of the experiment itself, and therefore we cannot assess how founder queens responded to decitabine at a molecular level. However, workers were certainly exposed to the effect of the chemical, and therefore a more collaborative behaviour by the treated workers (less dominance, more nursing activity, etc.) might lead to the development of a bigger brood by the queen. This finding is in contrast with the higher aggressiveness, dominance and reproductive behaviour exhibited by workers when exposed to decitabine in absence of the founder queen (7). In queen-right colonies, this behavior would translate into early disruption of the cooperation phase and more abundant male production at the colony level [35]. In our experiment we found no signs of an earlier competition point in treated colonies, and male production tended to be higher in control colonies. Consistent with these findings, we did not find any indication of worker aggressiveness or altered behavior, and our dissections did also not indicate differences in worker ovary development among groups. These two papers show that altering methylation in different social contexts (Queenright vs queenless) has different effects. This intriguingly mirrors the predictions for genomic imprinting in different social contexts as predicted by Queller and Strassmann [36]. DNA Methylation is a major component of the imprinting systems in mammals and flowering plants [37].

The better colony development observed here also appears to be in contrast to the findings of Ellers et al. [38], who suggested that decitabine has an overall anti-metabolic activity that leads to a decline of the physical condition of the insects, and therefore results in phenotypic differences among treatments that are not dependent on DNA methylation. The concentration of decitabine that was used in our experiments was the same as the one used by Amarasinghe et al. (7), who showed that this concentration had no deleterious effects on bumblebee workers. Administration of decitabine, or the solvent itself (acetic acid), had a large effect on queen survival and egg laying when it was administered immediately after queen start-up. Due to its antimicrobial activity, low concentrations of acetic acid are commonly used in the food industry as preserving agent, suggesting that addition of acetic acid could have affected the symbiotic gut flora of the queens [39, 40]. Such effects were not observed when the sugar water treatments were offered two weeks later. We hypothesize that post-hibernating queens represent a sensitive stage in the colony cycle in terms of survival [41] and therefore the applied treatments, or the resulting changes in gut flora, may have affected colony initiation or even caused death. Moreover, our data have shown that the use of decitabine translates into differences in methylation patterns between the two experimental groups, including processes related to oogenesis, which also contradicts a cytotoxic role of the compound.

Use of decitabine did not induce early queen production, indicating that altered methylation patterns did not affect caste determination. Queen production in *Bombus hypnorum* can be induced by exposing the last larvae instar to juvenile hormone [42]. However, exposition to juvenile hormone had no proven effect in *B. terrestris*, as caste is known to be determined very early in larval development [43, 44]. Caste determination in bumblebees has been classically assumed to be controlled by the action of queen pheromones and the endocrine profile of the larvae itself [43, 45]. Further research is needed to elucidate if endocrine differences might be impacted by differential methylation in larvae, and the efficacy of such mechanism compared to the direct exposition of larvae to queen pheromones [46].

### DNA methylation patterns in bumblebees

In invertebrates, levels of DNA methylation are much lower than in vertebrates [47, 48]. In insects, DNA methylation is concentrated in gene bodies and associated with more stable patterns of gene expression [48]. The average cytosine methylation (CpG) in the genome of honeybees is 0.7%, and much lower figures for the other methylation types (less than 0.2%) [49]. In *Bombus terrestris*, we found an average CpG methylation of 0.5% in the control group, and similar methylation rates for the other methylation types. These results largely correspond with recent findings in *Bombus terrestris audax,* where non-CpG methylation rates were 0.4 (CHG) and 0.5% (CHH) [15]. Compared to CpG methylation, however, CHG and CHH methylation were not particularly enriched in coding regions, which indicates that they have a minor regulatory role. Even though our experimental bees were treated to induce methylation differences, we found a very limited amount of loci with significant methylation levels for non-CpG types, which seems to corroborate that these types of methylation have a lower impact on gene expression.

Because we used founder queens from a pure genetic line, they do not differ (at least significantly) in their genetic information, making them excellent models to assess phenotypic effects of the methylation differences we experimentally induced. The wasp *Nasonia* has emerged as a model for DNA methylation studies in insects, due to its naturally inbred nature [30]. Despite the controlled variation at the genetic level, methylomes might still be subject to other sources of variation [50] and the stability and inheritance of methylomes has been subject to debate [51]. Despite this controversy, the latest evidence from honeybees suggests that even though gene body CpG methylation can oscillate during development, it is kept mostly invariable in the germline, likely to preserve function and methylation patterns over generations [49]. Experiments with *Nasonia* have also indicated stable inheritance of methylation status through generations [30]. This also agrees with recent findings that have shown high-inter-colony variation in methylation [15].

### Efficacy of decitabine to alter methylation patterns

Although decitabine clearly affected the methylation patters on callow workers, it was not obvious that there was a reduction in overall cytosine methylation. While Amarasinghe et al. (7) attributed this finding to an artefact of the detection technique (methylation sensitive AFLP, known as MSAP, [52], our WGBS data also indicated higher methylation levels for the treated bees. Decitabine has been recently used in wasps to manipulate methylation levels, and the resulting methylomes were analysed by WGBS [34]. This study also showed that the chemical does not work as a pure demethylation agent (thus visible in the global % of cytosine methylation), as it has been previously described (reviewed in [53, 54]). Considering that the effect of the drug seems to be context-dependent [34], future studies that aim to manipulate phenotypes by using decitabine should carefully check the effect of the compound at the molecular level using the best resolution available.

The topical administration of another DNA methyltransferase inhibitor such as RG108 on honeybee workers induced reduction in global levels of DNA methylation, which was confirmed by an ELISA-based methylation assay. Such overall reduction resulted in a phenotypic consequence for these workers, that showed increased lifespan ([55]). A similar methodology was previously used by Biergans et al. [56], who administered RG108 or Zebularine to the honeybee thorax, proving a significant decrease in global methylation levels -here measured by capillary electrophoresis- for both agents.

We detected higher rates of cytosines that were methylated along the genome in treated bees, and correspondingly a larger list of loci that were significantly methylated within the treated set of workers. Although we have not checked the expression of these loci, recent reports have associated WGBS with expression patterns, and found a negative correlation between methylation status of a given loci and levels of expression [15, 49]. Therefore, it is likely that treated bees showed higher expression levels of the differentially methylated genes. Our GO enrichment analyses were suggestive of a non-random effect of decitabine in the identification of DML related to oogenesis regulation and oocyte mRNA localisation. Interestingly, increased expression of these pathways would be in agreement with the positive response that the compound produced in terms of brood development and production of workers.

## Conclusion

There is increasing evidence that DNA methylation has a pronounced impact on the phenotype expressed by insects [57]. In the particular case of social insects, epigenetically-driven phenotypic differences may be particularly important, as they may affect the different roles that colony members with identical genetic information play within the colony organization. In our experiments, we have used an inbred line of bumblebee queen founders, and epigenetic differences were experimentally induced by adding the DNA methyl-transferase inhibitor decitabine to sugar provisions. Addition of the chemical resulted in altered DNA methylation patterns that led to a set of differentially methylated loci (with smaller methylation levels at the treated group) including some oogenesis. In contrast to previous research, queen production over worker production or early worker reproduction were not induced, and colonies showed a better cooperative behaviour, which led to higher worker production and less males. Considering that the genome of *Bombus* is rather simple and well annotated, more targeted work (targeted knock-downs) is needed to establish direct links between phenotypes (caste, reproduction) to causal CpG methylation on specific genes.

## Methods

### Experimental setup

A total of 24 lab-reared *B. terrestris* queens (Biobest Group, Westerlo, Belgium) from one single genetically pure, inbred line were used for all experiments. Males were obtained from separate colonies of the same original population, and they were discarded after one mating event. Fecundated queens that survived hibernation were set in separate cages and kept at 26 °C and 60% humidity on a diet of 50% v/v Biogluc® and Gamma irradiated honeybee collected, multifloral pollen ad libitum until they developed into full colonies.

A stock solution of 5-aza-20-deoxycytidine (decitabine) was made by dissolving 5 mg of decitabine (Sigma Aldrich, Belgium) in 2 ml of 1: 1 v/v acetic acid: distilled water solution. 10 mM decitabine was added to sugar water (0.0925% v/v) (7) and fed to two test groups of six colonies each at either one or three weeks after queen start-up (12 colonies in total). The control group (12 colonies) was fed with standard sugar water, plus the solvent (acetic acid), also at 0.0925% v/v. Solutions were provided to each colony on a weekly basis throughout the entire experiment. Neither the treatment nor the control colonies that received the sugar treatment from the first week after the start of the experiment did develop properly. Just 1 out of 6 queens receiving the treatment managed to lay eggs and start a colony, while this number was 0 out of 6 in the control group. As a result, these colonies were removed from subsequent analyses. Because similar effects were found for the control and treatment colonies, we conclude that the addition of the solvent to the sugar solution had a deleterious effect on the survival/fitness of queens shortly after hibernation. No such effects were observed when the feeding solutions were provided to the colonies two weeks later and all colonies followed the subsequent development stages. Exposure 2 weeks after queen start-up, as done in our experiment, still guaranteed that all resulting larvae were exposed to decitabine.

### Colony development and behavioral records

Colony development was monitored over a 10-week period. For each colony, we assessed the developmental time by determining the timing of first egg-laying, first pupation and first emergence of adults (workers, males). For each colony, we also counted the number of adult workers, pupae, larvae, dead larvae, and egg cups at week 8 and week 10. Counts at week 8 and 10 represent the colony development before and after the competition point, respectively. Counts of dead larvae at week 10 was not possible because it is nearly impossible to obtain accurate estimates in fully occupied nest boxes. Colony development was categorized into no development (no brood being produced), and successfully developed colonies, that follow the normal timing of development for the subsequent phases [19]. In addition, the behavior of the colony was recorded by observing colonies twice a week for 15 min throughout the trial. During these observations, we annotated queen dominance, worker aggressiveness as well as indications of worker reproduction (competition point), egg dumping, and nursing behavior. Egg dumping was expressed through bees placing abandoned eggs on top of the sugar water reservoir.

### DNA methylation on workers

After eight weeks, two colonies were randomly chosen per treatment. From each selected colony, up to five coetaneous adult workers were collected. Each worker was dissected using a fresh Ringers solution (Sodium chloride 2.25 g/L, Potassium chloride 0.10 g/L, Calcium chloride 6H_2_O 0.12 g/L, Sodium bicarbonate 0.05 g/L) to extract the brain tissue. At the same time, ovary development was assessed following the scale provided by [35], which we simplified to a three-level score of no development, intermediate and fully developed ovaries. DNA was then extracted from each flash-frozen brain sample using the EZNA Insect kit (Omega Bio-Tek), following the instructions from the manufacturer. DNA quality and quantity were determined by Nanodrop and Qubit® fluorospectrometers. Samples that yielded less than 1 μg DNA/μl were discarded from subsequent analyses.

### DNA extraction and library

Bisulphite conversion of genomic DNA combined with next-generation sequencing (WGBS) was used to measure the methylation state of the whole genome, the methylome, at single-base resolution [58]. Fourteen WGBS non-directional libraries (seven workers per treatment, taken from two colonies per treatment, with three to four workers per individual colony) were prepared by BGI Tech Solutions Co (Hong Kong). This involves DNA fragmentation, adapter ligation, bisulphite treatment, size selection and amplification. Resulting libraries (14, one per bee brain sample) were sequenced by using 100 bp paired-end bisulfite sequencing on a HiSeq 2000 machine (Illumina, Inc.) by BGI Tech Solutions Co.

### Data analyses

#### Colony development

To test whether colony development differed between founders that were assigned to different treatments, we used a Wilcoxon test with the number of days before egg laying, pupae appearance, male and worker emergence as dependent variables, while treatment was considered as fixed factor.

A generalized linear model with Poisson distribution was used to investigate whether brood size (the sum of egg cups and larvae) and the number of workers at eight and ten weeks after queen start-up differed between treatments. Treatment and counting week, as well as their interaction, were treated as fixed factors and brood size and the number of workers as dependent variables. Post-hoc analyses (Tukey HSD) were conducted to see whether the dependent variables differed significantly between treatments for each counting week. A similar model was used to investigate whether the number of dead larvae differed between treatments at week 8. Here treatment was the only fixed factor.

We calculated contingency tables to investigate whether the number of individuals assigned to the respective categories of ovary development differed between treatments. Significance was estimated using a Fisher’s exact test. Similar analyses were conducted to see whether dumping behaviour differed between treatments.

#### DNA methylation analyses

Poor quality reads/bases and adapter sequences were removed using the bbduk.sh function of BBMap (https://sourceforge.net/projects/bbmap/). Bismark v0.18.1 [59] was used to align reads to the *B. terrestris* genome (GCF_000214255.1 [60]) using the non-directional protocol,remove PCR duplicates, and filter non-converted reads. The Bismark BAM file output was processed using methylKit [61] to remove base calls with a quality below Q30 and filtered to remove low (< 10 reads) and high (> 99.9th percentile) coverage loci. This processed file was loaded in methylKit and filtered to exclude loci that were present in less than four samples per group. A binomial test was used to make per-loci methylation status calls, using a 1% error rate. Only loci with significant levels of methylation in at least one sample were subsequently tested for differential methylation between groups using the Chi-squared test in methylKit, controlling for colony as a covariate and correcting for overdispersion. A minimum methylation difference of 10% was necessary for a locus to be considered differentially methylated, using an FDR-adjusted *q*-value of 0.05. Differentially methylated loci (DML) were further curated manually and loci where the result was driven by a single methylated sample excluded.

Differentially methylated loci (DML) were extracted from the genome and annotated using a custom-made database [62]. GO enrichment was conducted against all RNA features in the bumblebee genome using GOStats [63] to conduct a hypergeometric test, with significant GO terms identified using Benjamini-Hochberg correction (adjusted *p*-value 0.05).

## Supplementary Information

**Additional file 1: Figure S1. (A)** Principal component Analyses (PCA) plot generated using methylKit function *PCASamples*, showing CpG methylation for the 7 control bee samples and the seven treated bee samples. **(B)**. Dendogram generated using methylKit function *clusterSamples* showing sample cluster by treatment. Red labels indicate control samples and blue labels represent decitabine-treated samples. Black labels in dendogram depict the colony of origin of each sample.

**Additional file 2.** Table 1 Overview of mapping efficiency, methylation rates in CpG, CHG and CHH contexts, number of significantly methylated loci and methylation fraction per loci for the 14 workers subjected to WGBS.

**Additional file 3.** Text files with data and code used for analyses. We provide datasets on colony developmental data (countings: ctres, temporal overview: events3c) and ovary development (ovarydev). Additional text files are available for the R code (R script) and WGBS analyses (supp_code).

## Data Availability

Sequence Read Archive (SRA) data analysed during the current study are available in the NCBI repository, https://www.ncbi.nlm.nih.gov/sra/PRJNA638377, in read-only format. Other data and scripts generated or analysed during this study are included in the submission as supplementary information files.

## Abbreviations

CHH: DNA sequence context characterized by cytosines followed by two consecutive H residues, where H correspond to Adenine, Thymine or Cytosine
CHG: DNA sequence context characterized by cytosines followed by H residues (which correspond to Adenine, Thymine or Cytosine) and Guanine
CpG: DNA sequence context characterized by Cytosines followed by Guanine residues
DML: Differentially Methylated Loci
DNA: DeoxyriboNucleic Acid
Dnmt1: DNA (cytosine-5)- methyltransferase 1
GO: Gene Ontology
mM: milimolar
mRNA: Messenger RiboNucleic Acid
WGBS: Whole Genome Bisulfite Sequencing
