# Supplementary material for: The effect of DNA methylation on bumblebee colony development
Source: BMC Genomics. 2021 Jan 22;22:73. doi: 10.1186/s12864-021-07371-1 (PMC7821684; doi:10.1186/s12864-021-07371-1)
Supplement: Supplementary file 1 — Additional file 1: Figure S1. (A) Principal component Analyses (PCA) plot generated using methylKit function PCASamples, showing CpG methylation for the 7 control bee samples and the seven treated bee samples. (B). Dendogram generated using methylKit function clusterSamples showing sample cluster by treatment. Red labels indicate control samples and blue labels represent decitabine-treated samples. Black labels in dendogram depict the colony of origin of each sample. [file 12864_2021_7371_MOESM1_ESM.docx]

**Figure S1. (A)** Principal component Analyses (PCA) plot generated using methylKit function *PCASamples*, showing CpG methylation for the 7 control bee samples and the seven treated bee samples. **(B)**. Dendogram generated using methylKit function *clusterSamples* showing sample cluster by treatment. Red labels indicate control samples and blue labels represent decitabine-treated samples. Black labels in dendogram depict the colony of origin of each sample.

| **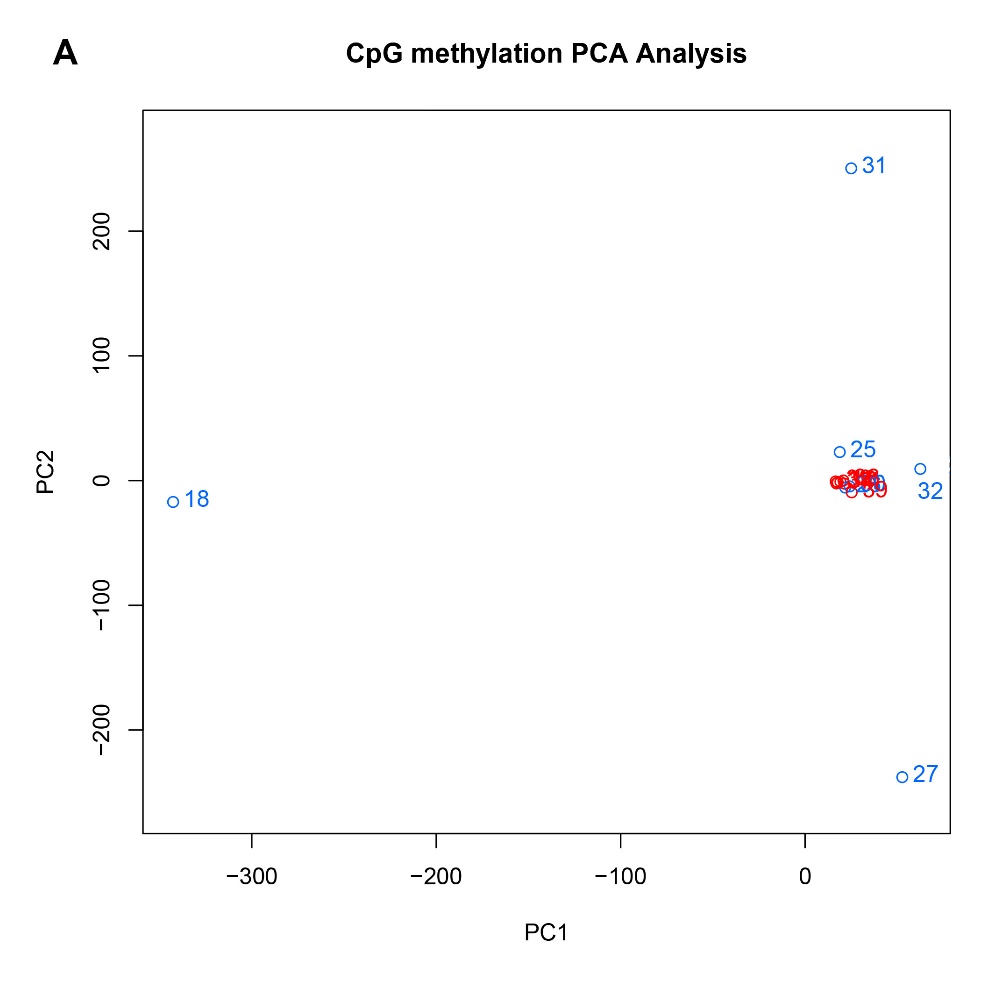** | **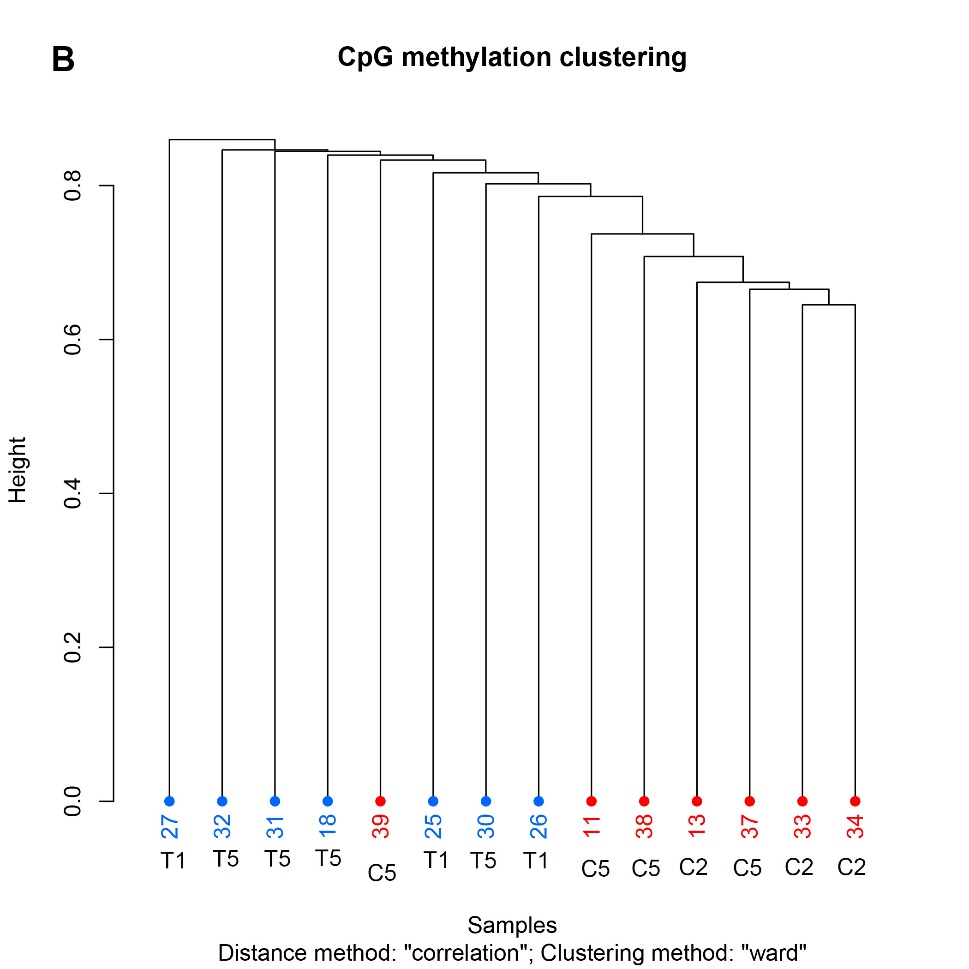** |
| --- | --- |
