# Supplementary material for: The effect of DNA methylation on bumblebee colony development
Source: BMC Genomics. 2021 Jan 22;22:73. doi: 10.1186/s12864-021-07371-1 (PMC7821684; doi:10.1186/s12864-021-07371-1)
Supplement: Supplementary file 2 — Additional file 2. Table 1 Overview of mapping efficiency, methylation rates in CpG, CHG and CHH contexts, number of significantly methylated loci and methylation fraction per loci for the 14 workers subjected to WGBS. [file 12864_2021_7371_MOESM2_ESM.pdf]

Hymenoptera: genetically identical specimens get a completely different phenotype

Q1: What is the role of DNA methylation on caste determination, worker reproductive behavior and overall colony development?

Lack of genetic clones! → Pure genetic line of founder queens

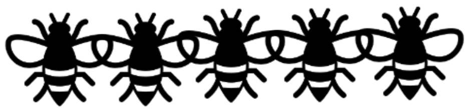

6 colonies per treatment

Follow up of colony development

Continuous administration of **methylation disruptor**

Week 3

Week 5

Week 8

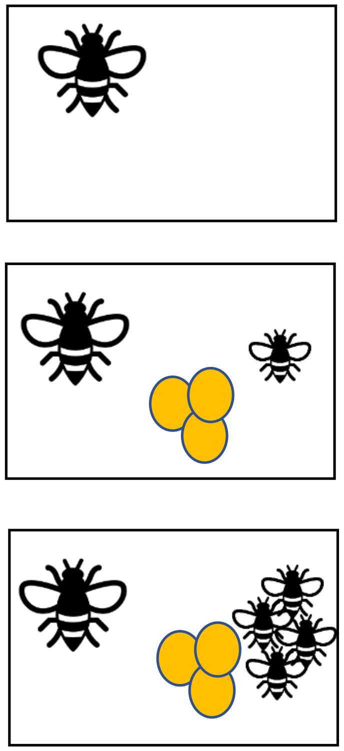

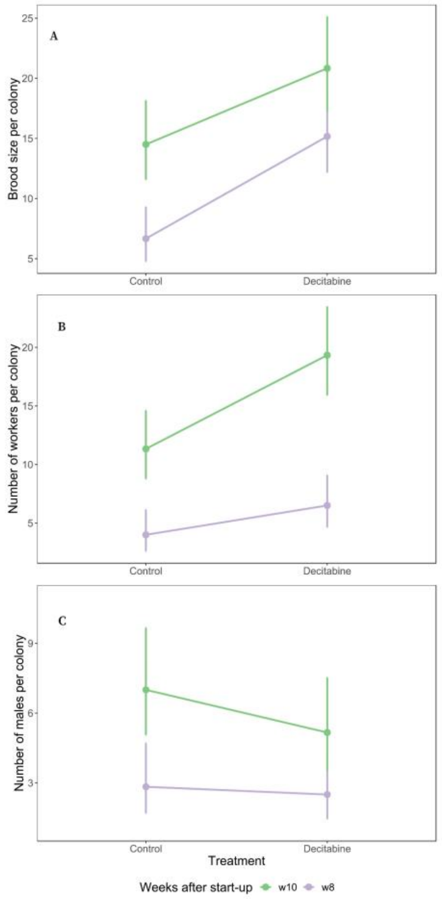

Control

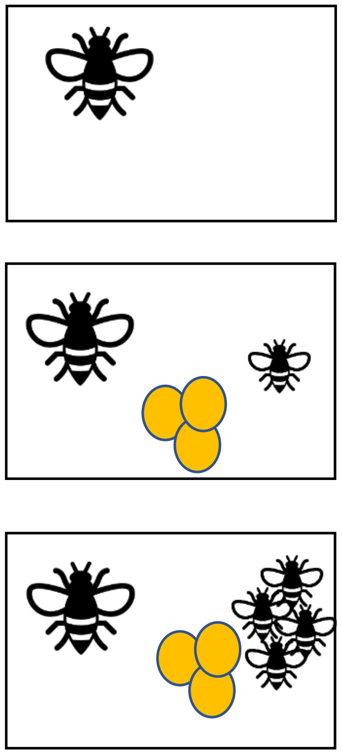

Increased worker production

Increased male production

Q2: What is the effect of Decitabine on genome methylation on our experimental subjects?

Random dissection of workers (brain tissue)

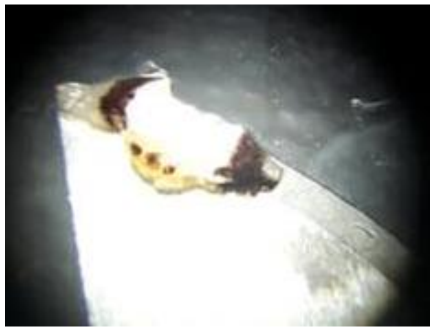

Whole genome bisulfite sequencing

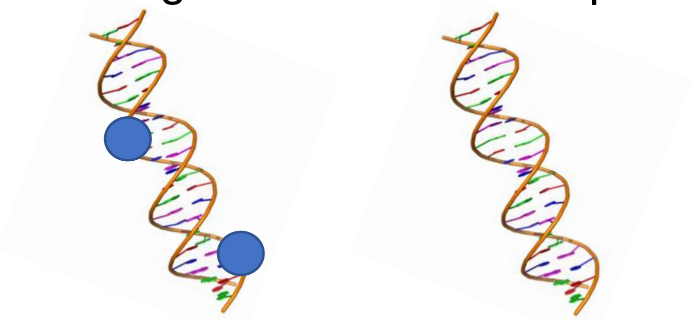

Methylated cytosines – Diff gene expression

Fewer methylated loci in control colonies

Where in genome?

→ CpG: 22 annotated loci

Which biological funtions are affected?

→ Neuron function, oocyte regulation and metabolic processes
