# Supplementary material for: The effect of DNA methylation on bumblebee colony development
Source: BMC Genomics. 2021 Jan 22;22:73. doi: 10.1186/s12864-021-07371-1 (PMC7821684; doi:10.1186/s12864-021-07371-1)
Supplement: Supplementary file 3 [file 12864_2021_7371_MOESM3_ESM.zip › supp_code.odt]

Data QC

BBMap v38.51

	bbduk.sh in1=1.fq.gz in2=2.fq.gz out1=cut.1.fq.gz 	out2=cut.2.fq.gz ftl=15 overwrite=t

	bbduk.sh in1=cut.1.fq.gz in2=cut.2.fq.gz out1=1.final.fq.gz 	out2=2.final.fq.gz ktrim=r 	ref=/cm/shared/apps/bbmap/38.51/	resources/adapters.fa k=23 mink=8 hdist=1 minlen=20 qtrim=rl 	trimq=20 tbo tpe overwrite=t

Alignment and post-alignment processing

Bismark v0.18.1

	bismark “${GENOME}” -1 1.final.fq.gz -2 2.final.fq.gz -o "$	{ALIGN}" --non_directional"

	deduplicate_bismark -p --bam "${ALIGN}"/sample.bam"

	filter_non_conversion -p --threshold 2 	"${ALIGN}"/sample .deduplicated.bam"

methylKit 1.12.0, R version 3.6.1

	processBismarkAln( location = bam.files,

		sample.id=sample.list,

		assembly="bter",

		treatment=c(0,0,1,1,1,1,1,1,1,0,0,0,0,0),

 		save.folder="methylkit_bam",

 		save.context=c("CpG","CHG","CHH"), read.context="none",

 		nolap=TRUE, mincov = 10,minqual = 30)

Differential methylation analysis

methylKit 1.12.0, R version 3.6.1

#load libraries

library(methylKit)

library(GenomicFeatures)

library(Rsamtools)

#define list of samples, covariates and input files

sample.list <- list("11","13","18","25","26","27","30","31","32","33","34","37","38","39")

covariates <- data.frame(colony=c("C5","C2","T5","T1","T1","T1","T5","T5","T5","C2","C2","C5","C5","C5"))

CpG.file.list <- as.list(list.files(input.path,full.names = T, pattern = "CpG.txt")) 

#### load data ####

CpGDB <- methRead(CpG.file.list, sample.id=sample.list,

 assembly="BEES",

 treatment=c(0,0,1,1,1,1,1,1,1,0,0,0,0,0),

 context="CpG",

 dbtype = NA,

 header = T, sep = "\t",

 dbdir = path)

#filter data - remove loci with low or high coverage

CpG.filterDB <- filterByCoverage(CpGDB,lo.count = 10,

                                  lo.perc = NULL,

                                  hi.count = NULL,

                                  hi.perc = 99)

#unite the samples into a single table containing all loci that are present in 4 or more samples per group

CpG.uniteDB=methylKit::unite(CpG.filterDB, destrand = TRUE, min.per.group=4L)

#binomial test to identify loci with significant methylation 

#extract the data from the united table

CpG.uniteDB.all <- getData(CpG.uniteDB)

#separate into individual samples – cytosine and coverage data

bin1 <- CpG.uniteDB.all[,5:6]

bin2 <- CpG.uniteDB.all[,8:9]

bin3 <- CpG.uniteDB.all[,11:12]

bin4 <- CpG.uniteDB.all[,14:15]

bin5 <- CpG.uniteDB.all[,17:18]

bin6 <- CpG.uniteDB.all[,20:21]

bin7 <- CpG.uniteDB.all[,23:24]

bin8 <- CpG.uniteDB.all[,26:27]

bin9 <- CpG.uniteDB.all[,29:30]

bin10 <- CpG.uniteDB.all[,32:33]

bin11 <- CpG.uniteDB.all[,35:36]

bin12 <- CpG.uniteDB.all[,38:39]

bin13 <- CpG.uniteDB.all[,41:42]

bin14 <- CpG.uniteDB.all[,44:45]

#define and run the binomial function on each dataset, testing number of cytosine calls at a loci against total reads

bt <- function(a, b, p) {binom.test(a, b, p, alternative="greater")$p.value}

CpG.allrows <- data.frame(CT=numeric(),Ccount=numeric(),pVal=numeric(),FDR=numeric(),row=numeric())

for (df in list(bin1, bin2, bin3, bin4, bin5, bin6, bin7, bin8, bin9, bin10, bin11, bin12, bin13, bin14)) {

 

	colnames(df) <- c("CT", "Ccount")

	df <- na.omit(df)

	df$pVal <- mapply(bt, df$Ccount, df$CT, 0.01)

	df$FDR <- p.adjust(df$pVal, method = "BH", n = length(df$pVal))

	df$row <- as.numeric(rownames(df))

	dfmeth <- subset(df, df$FDR < 0.05)

	CpG.allrows <- rbind(CpG.allrows, dfmeth)

}

#extract rows with significant methylation in at least one sample

CpG.unique.rows <- as.vector(as.numeric(unique(CpG.allrows$row)))

CpG.uniteDB <- methylKit::select(CpG.uniteDB, CpG.unique.rows)

CpG.DBdiff=calculateDiffMeth(CpG.uniteDB, test="Chisq", covariates=covariates, overdispersion="MN")

CpG.sig.diff=getMethylDiff(CpG.DBdiff,difference=10,qvalue=0.05)
